# What do we know about children living with HIV-infected or AIDS-ill adults in Sub-Saharan Africa? A systematic review of the literature

**DOI:** 10.1080/09540121.2016.1176684

**Published:** 2016-07-08

**Authors:** Rachel E. Goldberg, Susan E. Short

**Affiliations:** ^a^ Department of Sociology, University of California Irvine, Irvine, CA, USA; ^b^ Department of Sociology and Population Studies and Training Center, Brown University, Providence, RI, USA

**Keywords:** Children, HIV/AIDS, adult HIV infection, adult AIDS illness, OVC, HIV-affected, Africa

## Abstract

Millions of children in Sub-Saharan Africa live with adults, often parents, who are HIV-infected or ill due to AIDS. These children experience social, emotional, and health vulnerabilities that overlap with, but are not necessarily the same as, those of orphans or other vulnerable children. Despite their distinctive vulnerabilities, research aimed at understanding the situation of these children has been limited until very recently. This review summarizes the state of knowledge based on a systematic search of PubMed and Web of Science that identified 47 empirical research articles that examined either the population *prevalence* of children living with HIV-infected or AIDS-sick adults, or the *consequences* of adult HIV infection or AIDS illness for child well-being. This review confirms that this population of children is substantial in size, and that the vulnerabilities they experience are multi-faceted, spanning physical and emotional health and schooling. Mechanisms were examined empirically in only a small number of studies, but encompass poverty, transmission of opportunistic infections, care for unwell adults, adult distress, AIDS stigma, lack of social support, maternal breastfeeding issues, and vertical HIV transmission. Some evidence is provided that infants, adolescents, children with infected or ill mothers, and children living with severely ill adults are particularly vulnerable. Future research would benefit from more attention to causal inference and further characterization of processes and circumstances related to vulnerability *and* resilience. It would also benefit from further study of variation in observed associations between adult HIV/AIDS and child well-being based on characteristics such as age, sex, kinship, severity of illness, TB co-infection, disclosure, and serostatus awareness. Almost one-quarter of the studies reviewed did not investigate variation based on *any* of these factors. More nuanced understanding of the short- and long-term effects of adult HIV on children’s needs and circumstances will be important to ongoing discussions about equity in policies and interventions.

## Introduction

The umbrella terms “Orphans and Vulnerable Children” (OVC) and “HIV-affected children” are deliberately inclusive. They recognize the vulnerability of a broad range of children, among them children living in Sub-Saharan Africa (SSA) whose lives are disrupted by the HIV/AIDS epidemic, including through pediatric HIV/AIDS, the illness and/or death of family and household members, and impacts on communities (Bryant & Beard, 2016; Sherr et al., 2008, 2014). This broad recognition provides a basis for policies and programs to extend needed outreach to many. At the same time, effective outreach and useful program evaluation can be hindered without adequate attention to heterogeneity in children’s risks and needs. Identifying continuing gaps between need and provision requires being able to distinguish among children’s circumstances, so as to be able to identify their distinct vulnerabilities and provide the right mix of targeted interventions.

One group of HIV-affected children with distinct vulnerabilities are those living with HIV-infected or AIDS-ill adults. These children experience social, emotional, and health vulnerabilities that overlap with, but are not necessarily the same as, those of orphans or other vulnerable children (Foster, 2006; Sherr et al., 2014). For example, when they live in close proximity to adults who are AIDS-ill, children can face exposure to opportunistic infections such as tuberculosis, pneumonia, and diarrheal disease (Centers for Disease Control and Prevention, 2009). HIV–TB co-infection is particularly common, with at least one-third of people living with HIV globally also infected with latent TB (World Health Organization [WHO], 2015). For young children, TB and other infections can be mistakenly attributed to less serious common childhood illnesses (WHO, 2012). Older children are particularly vulnerable when they provide care for AIDS-ill adults (Cluver, Operario, Lane, & Kganakga, 2012a, 2013b; Kuo, Cluver, Casale, & Lane, 2014). Children of all ages experience the social, emotional, and financial stresses of living with an HIV-infected and/or HIV/AIDS-ill adult (Andrews, Skinner, & Zuma, 2005; Sherr et al., 2014), and these stresses may be compounded by experiences of HIV-associated stigma in their communities (Cluver, Gardner, & Operario, 2008, 2013a; Goldberg & Short, 2012).

Despite their distinctive vulnerabilities, research focused on the health and well-being of children living with HIV-infected or AIDS-ill adults has been limited until very recently. Moreover, global monitoring efforts on children have more often provided statistics on orphans, mother-to-child transmission, pediatric AIDS, and adolescent risk behavior, less frequently reporting on the population of children living with HIV-infected or AIDS-ill adults (UNICEF, 2010, 2013).

Accordingly, the goals of this review are to summarize the state of knowledge on this population and to identify future directions for research and policy. Focused attention on these children is as critical as ever. Global efforts have resulted in major expansions of HIV/AIDS treatment (PEPFAR, 2012). In 2014, treatment reached 10.7 million people in SSA, about 41% of all people living with HIV, compared to 100,000 in 2002 (UNAIDS, 2015). Progress is being made on prevention as well, although new infections are still far too common. In 2014, 1.4 million people were newly infected in SSA, most of them (∼1.2 million) adults (UNAIDS, 2015). The combination of reductions in HIV mortality, sustained population growth, and the persistent addition of newly infected adults suggests that the population of children living with adults who are HIV-infected will remain substantial in the near-term (Bongaarts, Buettner, Heilig, & Pelletier, 2008).

A systematic review of research on children living with HIV-infected and AIDS-ill adults provides critical perspective for discussions of equity. First, it provides detail on the distinctive circumstances and vulnerabilities of a particular group of children within the OVC umbrella. Second, it describes variation *among* this population, with regard to severity of adult illness, knowledge of status, kinship, age, sex, and community characteristics. Assessing equity and meeting the needs of all children requires a deeper understanding of such variations.

## Methods

**Figure 1.**
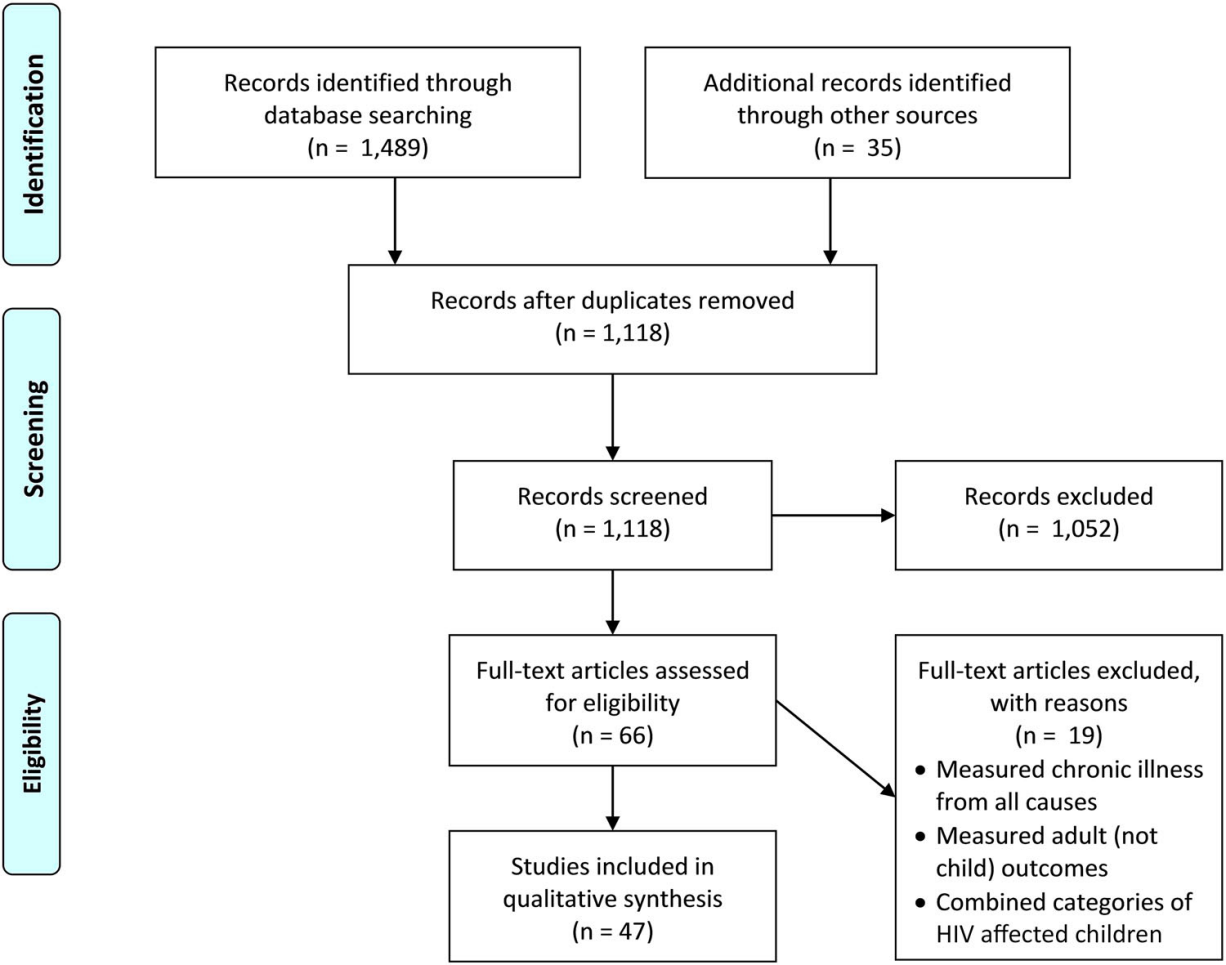
Flow diagram of search.

Articles were retrieved through PubMed and Web of Science searches. The searches were restricted to English language peer-reviewed articles published between 1 January 1990 and 1 March 2016, using a combination of the terms: HIV or AIDS, vulnerable children, HIV-affected children, and SSA. As depicted in Figure 1, which follows Preferred Reporting Items for Systematic Reviews and Meta-Analyses guidelines (Moher, Liberati, Tatzlaff, & Altman, 2009; Shamseer et al., 2015), these initial searches generated 1489 records. We identified 35 additional records through other sources (e.g., bibliographies, ad hoc searches). After removing duplicates, we were left with 1118 unique records to screen.

**Figure 2.**
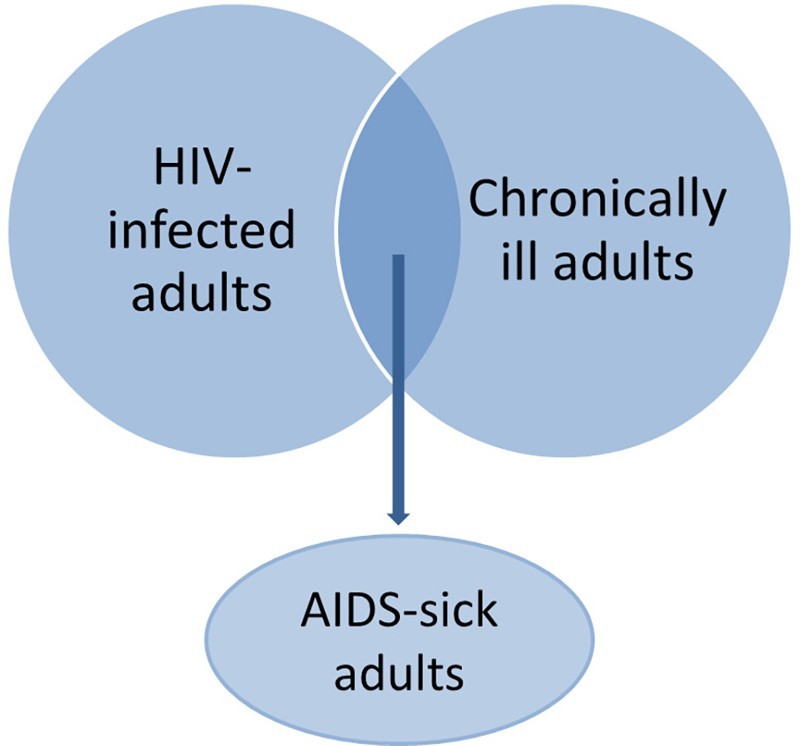
Measures of infection and illness used in analyses of HIV-affected children, and their overlaps.

We then examined titles and abstracts to identify articles for full-text review. We identified 66 empirical analyses (excluding reviews and opinion pieces) that appeared to investigate either the population *prevalence* of children living with HIV-infected or AIDS-sick adults, or the *consequences* of adult HIV infection or AIDS illness for child well-being. Upon further scrutiny, the articles depicted a range of adult experiences, including: (1) HIV infection; (2) chronic illness; and/or (3) HIV/AIDS-related sickness. These distinctions are important and summarized in Figure 2. Only some HIV-infected adults will be chronically ill, and not all chronic illness in high HIV-prevalence areas is related to HIV/AIDS. AIDS-sick adults are both HIV-infected and chronically ill. The final review includes only studies employing measures of adult HIV infection or AIDS sickness, and excludes studies that measure chronic illness generally.

Of the 66 articles assessed for eligibility, 19 were excluded because full-text review revealed that they (a) measured adult chronic illness broadly from all causes, (b) measured outcomes for adults rather than children, or (c) combined categories of HIV/AIDS-affected children, making it impossible to distinguish effects specific to the population of interest. The remaining 47 articles were subjected to detailed coding.

## Results

### Population prevalence of children living with HIV-infected or AIDS-ill adults

Studies that describe the population of children living with HIV-infected or AIDS-ill adults demographically are rare. One recent analysis of 23 SSA countries using Demographic and Health Survey (DHS) data (2003–2011) indicates that the percentage of children aged 0–17 years living with HIV-infected adults exceeds 10% in all Southern African countries and reaches as high as 36% (Short & Goldberg, 2015). A study focused on adolescents aged 15–17 years, which uses DHS data for 19 SSA countries (2003–2008), reports that roughly one-quarter live in households with infected adults in Lesotho, Zambia, and Zimbabwe, with the estimate for Swaziland reaching 43% (Magadi & Uchudi, 2015). Critically, DHS eligibility for testing and testing rates vary across settings, shaping these figures; nonetheless, these studies suggest that millions of children are living in households with HIV-infected parents in high-prevalence countries. Neither of these studies describes variation among children by age, gender, illness, or knowledge of serostatus. Short and Goldberg (2015) report on variation by kinship, finding that most children co-residing with tested, HIV-infected adults live with parents, often mothers, who are infected, although in some countries a substantial minority live with infected non-parental adults.

### Consequences for child well-being

#### Characteristics of studies reviewed

Supplementary table 1 provides detail on the 45 empirical studies that examine the consequences for child well-being of living with an HIV-infected or AIDS-sick adult. For each study, the table summarizes (1) data source and location; (2) study population and sample size; (3) study methodology; (4) outcomes measured; (5) the measure of adult HIV/AIDS employed (serostatus and/or illness); (6) consideration of variation based on child age, child sex, adult sex, kinship, severity of adult illness, adult knowledge of serostatus, and disclosure; and (7) attention to community-level factors. An expanded version of the table containing key relevant findings is available in the supplementary material. Each article in Supplementary table 1 was double-coded, with discrepancies in coding adjudicated by a third reviewer.

The 45 empirical studies in Supplementary table 1 span 26 SSA countries: Burkina Faso, Cameroon, Congo, Cote d’Ivoire, Democratic Republic of Congo, Gambia, Guinea, Ethiopia, Gabon, Ghana, Kenya, Lesotho, Liberia, Malawi, Mali, Mozambique, Niger, Rwanda, Senegal, Sierra Leone, South Africa, Swaziland, Tanzania, Uganda, Zambia, and Zimbabwe. Nine use data from nationally representative, population-based studies like the DHS. Seventeen base their results on community-based studies, and the remaining 19 rely on samples recruited from either health facilities (14) or non-governmental organizations (5). The articles span 29 unique data sources. The majority of the articles use quantitative methods (37), five employ exclusively qualitative methods, and three use mixed methods. Of the quantitative studies, 21 rely on data from cross-sectional studies and 16 use longitudinal data. Sample size varies greatly, ranging from *N* = 10 to *N*–55,749.

The outcomes examined in the 45 articles cluster in three general areas: child physical health and health risk behavior, emotional health, and education. The largest number of articles (28) include physical health or risk behavior outcomes, followed by emotional (15) and schooling outcomes (9).

#### Child physical health and health risk behavior

Twenty articles focus on under-5 health in particular. Longitudinal and cross-sectional studies observe excess infant and child mortality among children born to HIV-infected mothers compared to those born to uninfected mothers (Hong, Banta, & Kamau, 2007; Kuhn et al., 2005; Landes et al., 2012; Nakiyingi et al., 2003; Zaba et al., 2005). Studies able to distinguish children who are themselves HIV-infected reveal that HIV-exposed *uninfected* (HEU) children face higher risk of mortality compared to HIV-unexposed uninfected (HUU) children, particularly when maternal immunosuppression is high (Brahmbhatt et al., 2006; Fox et al., 2009; Schim van der Loeff et al., 2003).

With regard to under-5 morbidity, Marquez and colleagues identify higher risk of tuberculosis (2016) and other illnesses (2014) among HEU children compared to HUU children. However, Heinsbroek et al. (2016) find no significant difference in infant pneumococcal acquisition by maternal HIV status, and Landes et al. (2012) observe no significant differences in hospital admissions, early development, undernutrition, or functional limitations at 20 months between HEU and HUU children. Several longitudinal studies do reveal reduced infant growth (Makasa et al., 2007) and increased malnutrition (Marquez et al., 2014) among HEU children compared to HUU children, although two earlier longitudinal studies found little evidence of such differences (Bailey, Kamenga, Nsuami, Nieburg, & St. Louis, 1999; Sherry et al., 2000). Cross-sectional studies also show positive associations between maternal HIV status and under-5 malnutrition (Magadi, 2011a, 2011b; Mishra, Arnold, Otieno, Cross, & Hong, 2007).

Few of these studies examine mechanisms empirically. Makasa et al. (2007) implicate small size at birth and maternal subclinical mastitis in poor early growth among children with infected mothers. Marquez et al. (2014) point to early cessation of breastfeeding in explaining increased morbidity, although Fox et al. (2009) find that breastfeeding cessation accounts for only a small part of the influence of maternal HIV-related immunosuppression on child mortality. Less than half of the studies account for child HIV status, making it difficult in many cases to disentangle vertical transmission from other mechanisms.

Among older children, cross-sectional studies link parental and caregiver AIDS-related illness with self-reported illness and injury (Kidman, Hanley, Subramanian, Foster, & Heymann, 2010), adolescent HIV status (Kidman & Anglewicz, 2016), and severe pulmonary tuberculosis symptoms (Cluver, Orkin, Moshabela, Kuo, & Boyes, 2013b); tuberculosis risk is particularly high for children exposed to bodily fluids during provision of care to an AIDS-unwell adult. Wong et al. (2015) show using longitudinal data that living with an HIV-infected individual is also associated with higher risk of influenza-like illness and diarrhea for uninfected individuals compared to living in exclusively HIV-negative households.

The studies reviewed also show associations between living with HIV-infected and AIDS-ill adults and sexual risk behavior and abuse victimization. In cross-section, co-residence with HIV-infected adults is positively associated with having initiated sexual activity (Magadi & Uchudi, 2015). Cross-sectional (Cluver, Orkin, Boyes, Gardner, & Meinck, 2011) and longitudinal (Meinck, Cluver, & Boyes, 2015) studies also observe increased risk of physical and emotional abuse victimization among children living with AIDS-sick adults, with some evidence of mediation by poverty and adult disability (Meinck et al., 2015). Cluver et al. (2011) implicate food insecurity and exposure to abuse in associations between caregiver AIDS sickness and transactional sexual activity.

#### Child emotional health

The vast majority of articles examining child emotional health employ either qualitative data or cross-sectional quantitative data from community-based surveys. These studies link maternal and caregiver AIDS illness with increased internalizing and externalizing behaviors (Allen et al., 2014; Cluver, Orkin, Boyes, Gardner, & Nikelo, 2012b; Olang’o, Nyamongo, & Nyambedha, 2012; Palin et al., 2009; Sipsma et al., 2013), decreased adaptive functioning (Allen et al., 2014), and post-traumatic stress (Cluver et al., 2012b). Nonetheless, several qualitative studies of young carers make the point that caring for an ill loved one can also bring emotional benefits (pride, emotional bonding, emotional maturity), particularly if the child has access to other social resources that minimize the costs (Robson, Ansell, Huber, Gould, & van Blerk, 2006; Skovdal & Ogutu, 2009).

The studies reviewed reveal a number of potential mechanisms for associations between family HIV/AIDS and poor child emotional health, including parental distress (Allen et al., 2014; Lachman, Cluver, Boyes, Kuo, & Casale, 2014), HIV/AIDS stigma (Cluver et al., 2013a; Olang’o et al., 2012), lack of social support (Doku, Dotse, & Mensah, 2015; Skovdal & Ogutu, 2009), lack of positive parenting (Lachman et al., 2014), parent–child dysfunction (Allen et al., 2014), and poverty (Lachman et al., 2014). Disclosure of adult HIV status is presented as a pathway to both positive and negative psychosocial well-being. Qualitative findings suggest that secrecy leaves children, particularly adolescents, with feelings of resentment and anger (Withell, 2009; Wood, Chase, & Aggleton, 2006). Nonetheless, adults are often ill-equipped to handle children’s distress positively post-disclosure (Wood et al., 2006). Cross-sectional quantitative results by Palin et al. (2009) link caregiver disclosure with externalizing (but not internalizing) behaviors. Sipsma et al. (2013) find that compared to children who knew nothing about their mothers’ status, children who had been told *something* was wrong, without explicit mention of HIV, exhibited better behavior and adaptive functioning. When disclosure included mention of HIV, however, emotional outcomes were not distinguishable from children told nothing.

#### Education

Insights on the educational consequences of co-residing with HIV-infected or AIDS-ill adults derive primarily from cross-sectional quantitative studies and qualitative studies (Floyd et al., 2007; Pufall et al., 2014 are exceptions). Having an HIV-infected mother (Akbulut-Yuksel & Turan, 2013) or parent (Floyd et al., 2007; Mishra et al., 2007), or co-residing with any HIV-positive adults (Atwani Akwara et al., 2010), is associated with lower school attendance, especially in secondary school (Floyd et al., 2007). Similarly, living with an AIDS-ill caregiver or parent is associated with non-enrollment (Cluver et al., 2013a; Orkin, Boyes, Cluver, & Zhang, 2014) and non-attendance (Cluver et al., 2012a; Orkin et al., 2014). In addition, several studies link maternal infection or caregiver HIV/AIDS sickness with deficits in grade progression (Akbulut-Yuksel & Turan, 2013; Orkin et al., 2014), being hungry at school (Cluver et al., 2012a), and difficulties concentrating at school (Cluver et al., 2012a; Orkin et al., 2014). Articles examining schooling among young carers find that they are a group at particular risk of irregular school attendance and drop-out (Olang’o et al., 2012; Pufall et al., 2014; Robson et al., 2006).

With regard to mechanisms, poverty influences the ability of families to pay school fees and the need for children to work (Cluver et al., 2013a; Orkin et al., 2014). Other pathways identified include internalizing problems and concentration problems (Orkin et al., 2014), and provision of care to unwell adults (Olang’o et al., 2012; Pufall et al., 2014; Skovdal & Ogutu, 2009).

#### Variation by age, gender, kinship, illness, and serostatus awareness

Several studies model variation among children through sample stratification or inclusion of interaction terms. Child age and child sex were the most frequently considered axes of variation, incorporated into 21 and 14 articles, respectively. Several studies observe no significant variation by child age (e.g., Kidman et al., 2010; Lartey et al., 2014; Marquez et al., 2016), but those that do suggest that infants may be particularly vulnerable to physical health consequences (Magadi, 2011a, 2011b; Marquez et al., 2014; Nakiyingi et al., 2003), and adolescents to educational and emotional consequences (Floyd et al., 2007; Pufall et al., 2014; Wood et al., 2006). Many articles find no significant variation by child sex (Atwani Akwara et al., 2010; Floyd et al., 2007; Kidman & Anglewicz, 2016; Magadi & Uchudi, 2015; Orkin et al., 2014; Pufall et al., 2014). However, several show heightened vulnerability for under-5 boys with regard to nutrition (Magadi, 2011a, 2011b), and for adolescent girls with regard to sexual risk behavior and schooling (Cluver et al., 2013a; Olang’o et al., 2012; Robson et al., 2006). Studies of young carers also observe that while both girls and boys participate in caring, girls more often have nursing responsibilities (Olang’o et al., 2012; Robson et al., 2006).

A smaller number of the studies examine variation by severity of adult illness (8), disclosure (4), kinship (4), or adult sex (1), and none consider differentials by adult knowledge of serostatus. Associations between maternal HIV status and physical and emotional health are shown to be strongest when mothers are symptomatic, highly immunocompromised, and/or severely ill (Allen et al., 2014; Brahmbhatt et al., 2006; Fox et al., 2009; Kuhn et al., 2005; Sipsma et al., 2013; Zaba et al., 2005). As noted above, the studies present a mixed picture of whether disclosure of HIV status to children heightens or mitigates the emotional consequences of adult HIV/AIDS. With regard to kinship, most articles focus only on mothers or aggregate the status of all adult household members. Magadi (2011b) distinguishes the serostatus of mothers and other adult household members, and finds that only maternal HIV is significantly associated with child malnutrition. Similarly, Kidman et al. (2010) observe a significant link between co-residence with AIDS-sick parents and child health, but no independent effect of living with AIDS-sick non-parental relatives. Skovdal and Ogutu (2009) find that how closely related a care recipient is can influence how young carers cope with their duties. Assessing kinship and sex, Kidman and Anglewicz (2016) show that maternal, but not paternal, HIV status is associated with the odds of adolescent HIV infection.

#### Consideration of community factors

Eight studies consider community-level factors. Two (Magadi, 2011a, 2011b) model interactions between adult HIV infection and community variables, and find that links between maternal infection and child undernutrition are weaker in communities with higher HIV prevalence. Using community fixed effects analysis, Akbulut-Yuksel and Turan (2013) observe that high community HIV prevalence is associated with decreased schooling even in the absence of maternal infection; however, maternal HIV infection has a larger association with human capital development than community HIV prevalence. Kidman et al. (2010) use multi-level models and find no evidence of significant associations between community-level orphan prevalence or all-cause mortality and children’s health, independent of residence with AIDS-ill parents.

Other studies examine community factors as mediators. Cluver et al. (2013a) identify community violence and stigma as pathways between parental AIDS sickness and children’s psychological distress. Findings from qualitative studies also indicate the importance of community-level stigma (Olang’o et al., 2012; Skovdal & Ogutu, 2009).

## Discussion

The body of literature focused on SSA children who co-reside with HIV-infected or AIDS-ill adults, while growing rapidly, is small in volume relative to research focused on other OVC groups, such as orphans. This review establishes that (1) this population of children is substantial in size; (2) co-residing with an HIV-infected or AIDS-sick adult is associated with vulnerability; (3) this population of children has needs that may be distinct from those of other OVCs; and (4) children’s vulnerabilities vary by individual, household, and community characteristics.

Children’s vulnerability is multi-faceted. Living with an infected adult and/or with an adult experiencing AIDS-related illness is associated with a range of physical and emotional health outcomes in early and middle childhood and adolescence. It is also linked with children’s school enrollment, attendance, and progress. As voiced by others (e.g., Cluver et al., 2012a, 2012b), the strength of these associations, often even after controls for orphan status, suggests that the poor outcomes observed frequently among AIDS orphans may reflect a longer cycle of difficulties that began with parental infection. Mechanisms for these associations, which were examined empirically in only a small proportion of the reviewed studies and varied across outcomes, encompass poverty, care for unwell adults, adult distress, AIDS stigma, lack of social support, transmission of opportunistic infections, maternal breastfeeding issues, and vertical transmission. Although tentative, the studies also provide initial evidence of particularly vulnerable sub-groups, including infants, adolescents, children for whom the infected or ill adult is a mother, and children living with severely ill adults.

While the accumulated research yields important insights, further work remains. Future research would benefit from more attention to causal inference, more detailed investigation of population heterogeneity, and further characterization of processes and circumstances related to vulnerability *and* resilience. To more reliably assess causality and address concerns about selectivity and confounding (e.g., that other unobserved factors could be associated with both adults' likelihood of infection and their children’s health or schooling), we urge additional longitudinal research. Two-thirds of the quantitative studies reviewed were based on cross-sectional data. Moreover, virtually all of the studies were observational in nature. To isolate the particular pathways linking adult infection with child outcomes, intervention studies (including randomized controlled trials) may also be beneficial (Desmond et al., 2014). Additional qualitative research can also help elucidate pathways. In assessing pathways, we reiterate the call of others (e.g., Robson et al., 2006; Sherr et al., 2014; Skovdal, 2012) to investigate not only paths to vulnerability, but also to examine resilience-promoting factors among this population of children. We also suggest further exploration of the influence of community-level factors in shaping children’s outcomes.

In addition, we recommend further study of heterogeneity in observed associations between adult HIV infection and child well-being based on characteristics such as age, sex, kinship, severity of illness, and serostatus awareness. Almost one-quarter of the studies reviewed did not investigate variation based on *any* of these factors. Particularly sparse in the studies was examination of variation based on kinship, gender, and serostatus awareness. In addition, notably few studies considered HIV/TB co-infection and treatment, despite its significance in the region and its relevance to children’s health. Elaborating these variations may require more and better data, but such data will be important to ongoing discussions about equity in policies and interventions.

This review is not without limitations. The inclusion criteria included only articles in English and in peer-reviewed journals. Reports and monographs were excluded. We were also limited by the search terms we employed. Because there is not a single term used consistently for this population of children (unlike other HIV-affected groups like orphans), we kept our search as general as possible, and we supplemented the database searches with additional sources from the bibliographies of relevant articles. Finally, our review is also limited by bias in the publication of articles (e.g., articles with statistically significant results) and in the effect sizes of newly discovered non-null associations (Chan, Hrobjartsson, Haahr, Gotzsche, & Altman, 2004; Ioannidis, 2008; Song, Eastwood, Gilbody, Duley, & Sutton, 2000).

Finally, to assist us with interpreting our review, we examined peer-reviewed articles that evaluated programs targeted at OVCs for their results and recommendations related to support for children living in households with infected or AIDS-ill adults. This literature was most notable, however, for its absence of attention to this particular group of children. Numerous observers have called for more attention to family-centered care (e.g., Heymann, Clark, & Brewer, 2008; PEPFAR, 2012; Richter et al., 2009, 2010), and suggest that such care could offer needed support for children. We observe that integrating the distinct needs of children living with HIV-infected or AIDS-ill adults into both family-centered care and community outreach would support many children in high-prevalence areas, and deserves considered discussion as issues of equity in support for vulnerable children are addressed.
