# Supplementary material for: What do we know about children living with HIV-infected or AIDS-ill adults in Sub-Saharan Africa? A systematic review of the literature
Source: AIDS Care. 2016 Jul 8;28(sup2):130–41. doi: 10.1080/09540121.2016.1176684 (PMC4991228; doi:10.1080/09540121.2016.1176684)
Supplement: Supplementary Material.doc [file caic_a_1176684_sm7152.doc]

**Supplementary Table 1. Empirical articles examining consequences for child well-being of co-residence with an HIV-infected or AIDS-ill adult (Expanded)**

| **Author, year** | **Data sources, location** | **Study population, sample size** | **Study method** | **Outcomes measured** | **Measure (a)** | **Model variation by age, sex, kinship, illness, serostatus awareness, disclosure? (b)** | **Consider commun-ity-level factors?** | **Key relevant findings** |
| --- | --- | --- | --- | --- | --- | --- | --- | --- |
| Akbulut-Yuksel & Turan 2013 | DHS, 11 SSA countries (1991-2007) | Mothers aged 15-49, children 13-17; N=8,992 mother-child pairs | Cross-sectional surveys | Years of schooling attained, school attendance, progress at school | S | No | Yes | - Maternal HIV infection decreases intergenerational transfer of human capital  - Children with HIV-infected mothers less likely to attend school than children with HIV-negative mothers, show slower progress in school  - High HIV prevalence in community hinders children’s human capital development, but not as much as maternal HIV |
| Allen et al. 2014 | Baseline study for randomized clinical trial, two communities in Tshwane, South Africa | HIV-positive mothers of HIV-negative child aged 6-10; N=361 | Cross-sectional survey | Adaptive functioning, internalizing behavior, externalizing behavior | S, I | AI | No | - Maternal illness associated with children’s internalizing and externalizing behavior, daily living skills, and communication through parental distress and parent-child dysfunction |
| Atwani Akwara et al. 2010 | DHS and AIDS Indicator Surveys (AIS), 8 SSA countries (2003-2006) | Children aged 0-17 | Cross-sectional surveys | Wasting (age 0-4), school attendance (age 10-14), sex before age 15 (age 15-17) | S | CA, CS | Yes | - In two of five SSA countries assessed (Cameroon and Zimbabwe), having HIV-positive adult in household increases odds of child wasting  - In three of eight SSA countries (Tanzania, Uganda, Zimbabwe), having HIV-positive adult in household decreases odds of school attendance  - In one of seven SSA countries (Uganda), having infected adult in household predicts early sexual debut for girls and boys  - Community HIV prevalence independently associated with school attendance (four countries), early sexual activity (one country), and wasting (two countries) |
| Bailey et al. 1999 | Prospective cohort study, Kinshasa, Congo (1989-1992) | HIV-infected (N=68) and uninfected (N=190) children of infected mothers, uninfected children of uninfected mothers (N=256) | Longitudinal survey | Growth in length, weight, and weight-for-length | S, I | CA | No | - HIV-infected children were stunted, underweight, and wasted compared to uninfected children  - Uninfected children of infected mothers were similar at birth to infected, but by 3 months had same anthropometric characteristics as children of seronegative mothers  - Neither serostatus nor level of immune suppression, nor symptoms of HIV-related illness were significantly related to growth retardation |
| Brahmb-hatt et al. 2006 | Prospective study, rural Rakai District, Uganda (1994-1998) | HIV-infected (N=69) and uninfected (N=267) infants of infected mothers, uninfected infants of uninfected mothers (N=3128) | Longitudinal survey | Cumulative mortality at 12, 18, and 24 months | S, I | CA, AI | No | - HIV-infected infants had higher mortality at all ages compared with uninfected infants born to uninfected mothers  - Cumulative mortality of uninfected children born to HIV-infected mothers significantly higher than children born to uninfected mothers at 18 months  - High HIV viral load and low CD4 count in mothers associated with increased hazard of child mortality (not distinguishing infected and uninfected infants) |
| Cluver et al. 2011 | Follow-up of 2005 study, peri-urban communities, Cape Town, South Africa (2009) | Children aged 15-23; N=687 | Cross-sectional survey | Severe emotional, physical, and sexual abuse; transactional sex | I | CS | No | - Caregiver AIDS sickness predicts exposure to severe emotional and physical abuse and transactional sex, but not sexual abuse  - Being both AIDS orphaned *and* living with AIDS-sick caregiver predicts exposure to abuse and transactional sex over and above either only, particularly for girls  - Relationship between caregiver AIDS sickness and transactional sex driven by food insecurity, exposure to physical and emotional abuse |
| Cluver et al. 2012a | Community-based study, Western Cape, South Africa | Adolescents aged 10-20; N=659 | Cross-sectional survey, in-depth interviews | School attendance, hunger at school, focus at school | I | No | No | - Major themes from qualitative analyses: 1) missing or dropping out of school to care for unwell adults; 2) being hungry at school due to poverty; 3) concentration problems due to worry about sick person  - In quantitative analyses, living in home with AIDS-ill caregiver predicted all three outcomes compared to living in homes affected by other sickness and healthy homes |
| Cluver et al. 2012b | Study in peri-urban communities, Cape Town, South Africa (2005-2009) | Children aged 11-25 in 2009; N=723 | Longitudinal survey | Anxiety, depression, post-traumatic stress | I | No | No | - Caregiver AIDS sickness predicts increased depression, anxiety, and posttraumatic stress  - Young people affected by caregiver AIDS sickness *and* AIDS orphanhood show cumulative negative effects |
| Cluver et al. 2013a | Study in 6 districts in 3 South African provinces  (2009-2011) | Children aged 10-17; N=6002 | Cross-sectional survey | Psychological distress, educational access, sexual health | I | CA, CS | Yes | - No direct association between parental AIDS illness and outcomes of interest  - Parental AIDS-sickness associated with AIDS stigma, which raises likelihood of child abuse, leading to psychological distress  - Parental AIDS-sickness associated with increased poverty, which is associated with stigma, abuse, inability to afford school fees, community violence  - In all models, parental AIDS-illness displayed stronger associations and more risk pathways than AIDS-orphanhood, especially via poverty and parental disability  - Girls in AIDS-affected families experience heightened vulnerability |
| Cluver et al. 2013b | Study in 6 districts in 3 South African provinces  (2009-2011) | Children aged 10-17; N=6002 | Cross-sectional survey | Severe pulmonary tuberculosis symptoms | I | No | No | - Caregiver HIV/AIDS illness associated with tuberculosis symptoms  -Exposure to body fluids during care provision to AIDS-unwell adult associated with large increase in symptoms  - Risk for TB exacerbated by overcrowding and food insecurity |
| Doku et al. 2015 | Study in Lower Manya Krobo District, Ghana | Children aged 10-17; N=291 | Cross-sectional survey | Perceived social support | S* | No | No | - Living with HIV-infected parent associated with lower levels of social support compared with non-OVCs, AIDS-orphaned children, and other orphaned children |
| Floyd et al. 2007 | Retrospective cohort study, Karonga District, Malawi (late 1980s-2000) | Children (<18 at baseline) of HIV-positive (N=487) and HIV-negative (N=1493) individuals | Longitudinal survey | Primary and secondary school attendance | S | CA, CS | No | - Among children <15 years old, educational achievement among children of HIV-positive and HIV-negative parents similar  - Parental HIV adversely associated with secondary school attendance |
| Fox et al. 2009 | Randomized controlled trial (RCT), Lusaka, Zambia (2001-2004) | HIV-uninfected infants (N=355) born to HIV-infected mothers | RCT | Child mortality through 18 months | S, I | AI | No | - Uninfected infants of mothers with CD4 counts <200 were breastfed for shorter durations and at increased risk of death through 18 months compared to infants with mothers with counts >500  - Small part of association between CD4 count and child mortality mediated through breastfeeding cessation |
| Heinsb-roek et al. 2016 | Study of pregnant women and infants, Karoga District, Malawi (2009-2011) | HIV-infected (N=54) and uninfected (N=131) mother/ infant pairs | Longitudinal survey | Pneumo-coccal acquisition | S | CA | No | - No significant difference in pneumococcal acquisition by maternal HIV status, even though pneumococcal prevalence was higher in mothers with HIV than mothers without   -Other children under age 5 in the households were the main transmitters to infants (not mothers) |
| Hong et al. 2007 | Ghana DHS (2003) | Children aged 0-59 months; N=3639 | Cross-sectional survey | Infant mortality | S | No | No | - Having HIV-infected mother associated with higher likelihood of dying before 12 months compared to having uninfected mother |
| Kidman & Angle-wicz 2016 | DHS data, 19 SSA countries (2003-2011) | Adolescents 15-17 years with HIV test results; N=43,289 | Cross-sectional surveys | Adolescent HIV status | S | CS, K, AS | No | - Independent of orphan status, male and female adolescents with HIV-infected mothers (but not HIV-infected fathers) more likely than those without to test positive for HIV infection |
| Kidman et al. 2010 | Malawi Integrated Household Survey (2004-2005) | Children aged 6-17; N=16,139 | Cross-sectional survey | Illness/injury last 2 weeks, illness/injury that impeded normal activities, chronic illness | I | CA | Yes | - Children 6-9 living with AIDS-ill parents had higher odds of reporting poor health, across all outcomes (including acute and chronic morbidity)  - Same pattern observed in children 10-17 |
| Kuhn et al. 2005 | Randomized controlled trial in Lusaka, Zambia (2001-2004) | HIV-uninfected infants born to HIV-infected mothers; N=620 | Longitudinal survey | Child mortality, hospital admissions, infant weight through 4 months | S, I | AI | No | - HIV-related immunosuppression (CD4 counts <350, high viral load) associated with higher risk of death and hospitalization, lower infant weight |
| Lachman et al. 2014 | Study in one urban and one rural community, KwaZulu Natal, South Africa (2009-2010) | Primary caregivers 18+ (N=2477) and one child 10-17 under their care (N=2477) | Cross-sectional survey | Positive parenting | I | No | No | - Caregiver illness associated with less positive parenting  - Mediated by poverty, caregiver depression, child behavior problems |
| Landes et al. 2012 | Retrospective cohort study, Zomba District, Malawi (2008) | Births to HIV-infected (N=173) and uninfected (N=214) mothers | Longitudinal survey | Deaths 0-20 months; hospital admission, delayed development, undernutriti-on, functional restrictions at 20 months | S | CA | No | - Exposure to HIV associated with higher risk of child death by 20 months  - At 20 months, increased risk of poor health outcomes among surviving HIV-infected children compared to HIV-unexposed children  - No significant differences at 20 months in health outcomes between surviving HIV-unexposed children and children who were HIV-exposed *but uninfected* |
| Lartey et al. 2014 | RIING study, Eastern Region, Ghana (2003-2008) | Pregnant women >18; N=552 | Longitudinal survey | Infant weight-for-age, weight-for-length, length-for-age | S | CA, CS | No | - Maternal HIV status associated with reduced infant growth in weight and length throughout first year of life |
| Magadi 2011a | DHS data, 18 SSA countries (2003-2008) | Children < 5 with HIV-infected mothers; N=3157 | Cross-sectional surveys | Stunting, wasting, underweight | S | CA, CS | Yes | - Risk of malnutrition among children of infected mothers particularly high among 1-year-olds, boys, those smaller than average at birth, those whose mothers had no education, those in poorest or single parent households  - Benefit of breastfeeding not evident among children with infected mothers  - No evidence that community-level HIV prevalence associated with child malnutrition  - Risk of malnutrition among children with infected mothers lower in countries with higher HIV prevalence |
| Magadi 2011b | DHS data, 18 SSA countries (2003-2008) | Children < 5; N=55,749 | Cross-sectional surveys | Stunting, wasting, underweight | S | CA, K | Yes | - Children with HIV-infected mothers more likely to be stunted, wasted, underweight compared to children with uninfected mothers  - Nutritional status of children 1-5 in households where other adults besides mother are infected not significantly different from those in households where no adult infected  - Link between maternal infection and stunting weaker in communities with higher HIV prevalence  - Link between maternal infection and wasting weaker in countries with higher HIV prevalence |
| Magadi & Uchudi 2015 | DHS data, 19 SSA countries (2003-2008) | Children aged 15-17 years; N=22,620 | Cross-sectional surveys | Ever sexual intercourse | S | CS | No | - Adolescents in households with infected adult have higher odds of having initiated sexual activity compared with those in households with no infected adult  - No evidence of gender variation, or variation across countries or regions, in association between co-residence with infected adult and sexual activity |
| Makasa et al. 2007 | Cohort study, Lusaka, Zambia (2001-2003) | Infants with no HIV symptoms at age 2-4 born to infected (N=85) and uninfected (N=184) mothers | Longitudinal survey | Infant weight and length | S | No | No | - Infants of HIV-infected mothers have poorer early growth than HIV-unexposed counterparts  - Major contributors to this were small size at birth and maternal subclinical mastitis |
| Marquez et al. 2014 | Secondary data from chemoprevention trial in Uganda (2010-2011) | HIV-unexposed uninfected (N=389) and HIV-exposed uninfected (N=186) children 6-24 months old | Longitudinal survey | Hospitalization, severe febrile illness, severe diarrhea, malaria, malnutrition | S | CA | No | - Non-breastfeeding HEU children 6-11 months-old had higher risk of hospitalizations, severe febrile illness, severe diarrhea, severe malnutrition compared with non-breastfeeding HUU  - No difference in morbidity outcomes between breastfeeding HEU and HUU aged 6-11 months  - In 12-24 month age group, increased risk of severe malnutrition for HEU among non-breastfeeding children |
| Marquez et al. 2016 | Study of mothers and children recruited from cohort study in Uganda (2013) | HIV-infected (N=149) and uninfected (N=151) mothers and uninfected children (N=476) 0-60 months old | Cross-sectional survey | Active and latent tuberculosis infection | S | CA | No | - HIV-exposed uninfected children had higher odds of TB infection compared to HIV-unexposed uninfected children  - Maternal TB predicts TB infection in children |
| Meinck et al. 2015 | Study in rural and urban locations of Mpumalanga and Western Cape, South Africa (2010-2012) | Children aged 10-17; N=3401 | Longitudinal survey | Child physical and emotional abuse victimization | I | CS, AI | No | - Children in AIDS-ill families at higher risk of physical abuse victimization; mediated through poverty and disability  - For boys, positive direct and indirect effect of AIDS-illness on emotional abuse through poverty  - For girls, positive indirect effect through poverty  - Children in families with other chronic illness at lower risk for abuse victimization, unless subject to higher levels of household disability |
| Mishra et al. 2007 | Kenya DHS (2003) | Children aged 0-4 (N=2,756) and 6-14 (N=4,172) in households selected for HIV testing | Cross-sectional survey | School attendance (6-14); stunting, underweight, wasting (0-4); vaccines (1-4); treatment for ARI and diarrhea (0-4) | S | CA | No | - Children of HIV-positive parents less likely to be attending school than children of HIV-negative parents  - Children of HIV-positive parents more likely to be underweight, less likely to receive treatment for ARI and diarrhea |
| Nakiyingi et al. 2003 | Cohort study, Masaka District, Uganda (1989-2000) | Births between 1989 and 2000 (N=3727) | Longitudinal survey | Infant and child mortality | S | CA | No | - Infant and child mortality risk higher for HIV seropositive than seronegative mothers  - Maternal HIV status showed interaction with child’s age, with stronger HIV hazard in infancy |
| Ndirangu et al. 2011 | Study in western Kenya (2006) | Children <5 living in HIV-affected (N=102) and unaffected (N=99) households | Cross-sectional survey | Stunting, wasting, underweight | S | CA | No | Prevalence of stunting (but not wasting or underweight) significantly higher in households with HIV-positive adults, compared to households with orphans only, households with HIV-positive adults and orphans, and HIV -unaffected households |
| Olang’o et al. 2012 | Ethnographic study, Bondo District, Kenya (2006) | Children aged 10-18 (N=19) and the people living with HIV whom they cared for | In-depth interviews, focus groups |  | I | CS, D | Yes | - Children became involved in caregiving due to lack of responsible adult to perform the role, which may be result of stigma/rejection by extended family members and neighbors  - Caregiving engendered psychological distress, physical burden, school drop-out, participation in wage labor, early marriage, transactional sex  - Girls and boys participated in caregiving; however, in households with both boys and girls, girls more often left with nursing duties |
| Orkin et al. 2014 | Study in peri-urban settlements around Cape Town, South Africa (2009) | Youth aged 11-25; N=723 | Cross-sectional survey | School enrollment, attendance, grade progression, concentration at school | I | CS | No | - Caregiver HIV/AIDS-sickness indirectly associated with non-enrolment and concentration problems via internalizing problems and combination of poverty and internalizing problems  - Caregiver HIV/AIDS-sickness indirectly associated with non-attendance through poverty, internalizing problems, and combination of the two  - Path from HIV/AIDS-sick caregiver to grade progression via concentration problems and combination of poverty and internalizing problems |
| Palin et al. 2009 | Study in three communities, Pretoria, South Africa (2004-2005) | HIV-infected mothers 24-29 who were primary caregivers of a child aged 11-16; N=103 | Cross-sectional survey | Internalizing and externalizing symptoms | S, I | AI, D | No | - Disclosure to children significantly predicted externalizing, but not internalizing, behaviors.  - Mother-child relationship quality and mother-co-parent relationship quality had direct but not interactive effects with disclosure on child functioning |
| Pufall et al. 2014 | Cohort study, Eastern Zimbabwe (1998-2011) | Children 6-24; N=10,000 for adult reports; N=5,520 for child reports (2009-2011 only) | Longitudinal survey | Primary/ secondary school attendance, grade for age, primary school completion, at least five “0” level passes | S | CA, CS, | No | - Young carers (boys and girls) less likely to attend secondary school, but not primary school  - No evidence that parental HIV status significantly influences children’s education outcomes |
| Robson et al. 2006 | Studies in rural and urban areas in Lesotho, Tanzania, and Zimbabwe | N=100 children 10-17 (Lesotho); N=270 children (Tanzania); N=9 young carers (Zimbabwe) | Cross-sectional surveys, in-depth interviews, storyboards | Schooling, psychosocial wellbeing | I | CS | No | - Caregiving has emotional costs and benefits; benefits include emotional bonding with relative, pride in caring, gain in maturity  - Girls do more caring than boys, but boys are sometimes young carers  - Caring for sick relative is more frequent explanation for irregular school attendance than for dropping out  - Girls’ schooling suffers more than boys’ |
| Schim van der Loeff et al. 2003 | 5-8 years’ follow-up of cohort of children in the Gambia (1993-2001) | Children born to HIV-infected and uninfected mothers; N=774 | Longitudinal survey | Child mortality through 6-8 years | S, I | CA | No | - Mortality hazards ratio of HIV-1 and HIV-2 infected children significantly increased compared to children of seronegative mothers  - Mortality hazards ratio of HIV uninfected children of infected mothers not significantly increased compared to children of seronegative mothers, overall. However, in analyses excluding first 4 months, mortality hazards significantly higher. |
| Sherry et al. 2000 | Cohort study, Nairobi, Kenya (1991-1994) | Children born to HIV-positive (N=234) and HIV-negative (N=139) mothers | Longitudinal survey | Length-for-age, weight-for-height | S | No | No | - No major differences in growth between seropositive children born to seropositive mothers, seronegative children born to seropositive mothers, and children born to seronegative mothers |
| Sipsma et al. 2013 | Baseline study for randomized control trial, Tshwane, South Africa (2006-2009) | Mothers who are primary caregivers of a child aged 6-10; N=509 | Cross-sectional survey | Adaptive functioning, externalizing behavior, internalizing behavior | S, I | AI, D | No | - Children of HIV-infected mothers whose mothers were symptomatic exhibited greater internalizing and externalizing behaviors compared with children of HIV-negative mothers  - Not true for children of asymptomatic mothers  - Compared to children who were not told anything about their mothers’ HIV status, children who were told something was wrong, without explicit mention of HIV, had better behavior and adaptive functioning. When disclosure included mention of HIV status, there were no significant differences from children not told anything. |
| Skovdal & Ogutu 2012 | Participatory action research project, Bondo District, Kenya | HIV-affected and caregiving children aged 12-17; N=48 | PhotoVoice | Peer social capital | I | No | No | - Schools provide children with useful platform to establish and draw on mix of friendship structures  - Children help each other through sharing and practical support, demonstrating that children are able to accumulate and benefit from ‘peer social capital’ |
| Skovdal & Ogutu 2009 | Participatory action research project, Bondo District, Kenya | Children aged 11-17 who care for adults ill with HIV/AIDS; N=48 (3 case studies) | PhotoVoice, in-depth interviews, focus groups | Psychosocial wellbeing | I | K | Yes | - Meanings young caregivers attach to their circumstances impacts how well they cope  - Symbolic and socioeconomic resources stemming from familial and social environments can allow children to draw on more positive aspects of caregiving  - Children’s previous experiences of caregiving and who they currently care for also influence the meanings they attach |
| Withell 2009 | Qualitative retrospective study, eastern Uganda | Parentally-bereaved adolescents aged 13-19; N=10 | Unstructured interviews | Psychosocial needs of AIDS-affected adolescents | I | D | No | - Adolescents’ level of awareness about parental illness affected their psychological needs  - Four awareness states: concealed reality, disguised reality, discerned reality, disclosed reality |
| Wong et al. 2015 | Population-based surveillance, Kibera slum, Nairobi, Kenya | HIV-negative household contacts of infected individuals (N=1830), individuals in HIV-negative households (N=13,677) | Longitudinal survey | Influenza-like illness (ILI), diarrhea, nonspecific febrile illness, burns | S | CA | No | - For individuals aged 5+, incidence of ILI and diarrhea was significantly increased in HIV-negative household contacts of HIV-infected individuals, compared with exclusively HIV-negative households  - Risk of ILI and diarrhea among HIV-negative persons directly proportional to number of HIV-infected persons in the home  - No increased rates of illness in children <5 who lived with HIV-infected individuals |
| Wood et al. 2006 | Qualitative study in six sites in Zimbabwe | Children aged 7-22 (N=56), adults (N=41), households (N=18) | Case studies, semi-structured interviews |  | I | CA, CS, D | No | - Disclosure within households was exception rather than norm  - Teens desired direct communications with adults about parental illness and death  - Secrecy left teens with feelings of resentment, broken trust, anger  - Adults often ill-equipped to identify and manage children’s distress positively |
| Zaba et al. 2005 | Two prospective cohort studies (Tanzania, Uganda), retrospective study (Malawi) | Births to mothers tested for HIV; N=10,849 | Longitudinal survey | Infant and child mortality | S | CA, AI | No | - Excess risk of mortality associated with having HIV-positive mother; effect persists until at least age three  - Even higher excess mortality risk associated with impending or recent maternal death |

Abbreviations: (a) Measure of adult HIV/AIDS at individual level: S=HIV serostatus, I=HIV/AIDS illness; (b) Does the study examine variation by…? CA=child age, CS=child sex, AS=adult sex, K=kin relationship of adult to child, AI=severity of adult illness, AK=whether adult knows he/she is infected; D=disclosure to child

*Measure is self-report of HIV status rather than biological measure of serostatus
